# Potentially Functional Variants of *DCTD* and *ENTPD2* in the Metabolism of Nucleotide Pathway Genes Predict Survival of HBV-Related Hepatocellular Carcinoma Patients

**DOI:** 10.3390/cancers18142253

**Published:** 2026-07-14

**Authors:** Yan Mao, Qiuling Lin, Yingchun Liu, Xiaoxia Wei, Zihan Zhou, Qiuping Wen, Yanji Jiang, Peiqin Chen, Xiumei Liang, Yuying Wei, Qingyi Wei, Wenjing Zhou, Hongping Yu

**Affiliations:** 1 Department of Experimental Research, Guangxi Medical University Cancer Hospital, 71 Hedi Road, Qingxiu District, Nanning 530021, China; maoyan.dr@sr.gxmu.edu.cn (Y.M.); liuyingchun@stu.gxmu.edu.cn (Y.L.); h1999206@sr.gxmu.edu.cn (Y.W.); 2 Department of Clinical Research, Guangxi Medical University Cancer Hospital, Nanning 530021, China; linqiuling@stu.gxmu.edu.cn (Q.L.);; 3 Key Laboratory of Cancer Molecular Medicine of Guangxi, 22 Shuangyong Road, Qingxiu District, Nanning 530021, China; 4 Department of Cancer Prevention and Control, Guangxi Medical University Cancer Hospital, Nanning 530021, China; zhouzihan@stu.gxmu.edu.cn; 5 Institutional Review Board Office, Guangxi Medical University Cancer Hospital, Nanning 530021, China; jiangyanji@sr.gxmu.edu.cn; 6 Editorial Department of Chinese Journal of Oncology Prevention and Treatment, Guangxi Medical University Cancer Hospital, Nanning 530021, China; chenpeiqin@stu.gxmu.edu.cn; 7 Department of Disease Process Management, Guangxi Medical University Cancer Hospital, Nanning 530021, China; liangxiumei@stu.gxmu.edu.cn; 8 Cancer Prevention and Treatment Research Institute, Greater Bay Area Institute of Precision Medicine, 6 Nanjiang 2nd Road, Nansha District, Guangzhou 511462, China; weiqingyi@ipm-gba.org.cn; 9 Department of Epidemiology, School of Public Health, Fudan University, 130 Dong’an Road, Shanghai 200032, China; 10 Human Resources Department, Guangxi Medical University Cancer Hospital, 71 Hedi Road, Qingxiu District, Nanning 530021, China; 11 Key Laboratory of Early Prevention and Treatment for Regional High Frequency Tumor, Guangxi Medical University, Ministry of Education, Nanning 530021, China; 12 Guangxi Key Laboratory of Early Prevention and Treatment for Regional High Frequency Tumor, Guangxi Medical University Cancer Hospital, Nanning 530021, China; 13 Guangxi Zhuang Autonomous Region Molecular Medical Engineering Research Center for Cancer, Nanning 530021, China

**Keywords:** nucleotide metabolism, genetic variants, hepatitis B virus, hepatocellular carcinoma, survival

## Abstract

**Purpose**: Nucleotide metabolism plays a critical role in cancer development, but the prognostic significance of genetic variants in nucleotide metabolism genes for hepatitis B virus (HBV)-related hepatocellular carcinoma (HCC) patients remains unclear. **Methods**: We performed Cox proportional hazards regression analyses to evaluate the association between genetic variants in 94 nucleotide metabolism-related genes and overall survival (OS) in 866 HBV-HCC patients. To assess the potential biological relevance of the identified variants, the Bayesian false discovery probability and false-positive report probability were applied for multiple testing correction. **Results**: Two independent SNPs, *DCTD* rs17074255 G>A (HR = 1.22, 95% CI: 1.06–1.40, *p* = 0.005) and *ENTPD2* rs3763662 G>A (HR = 1.18, 95% CI: 1.03–1.34, *p* = 0.015), were significantly associated with OS. A significant dose-dependent association between the number of risk genotypes and poorer OS was observed (*P*_trend_ < 0.001). Luciferase reporter assays demonstrated allele-specific regulatory effects of rs3763662 on *ENTPD2* expression (*p* < 0.001). *DCTD* and *ENTPD2* mRNA expression levels were significantly elevated in HCC tumors in the UALCAN database and in our 103 paired samples. Higher expression levels of both genes were associated with poorer survival in the TCGA cohort (*p* = 0.003 and *p* < 0.001). **Conclusions**: Our findings suggest that *ENTPD2* rs3763662 (supported by direct functional evidence) and *DCTD* rs17074255 (supported by eQTL and expression associations) may serve as potential prognostic indicators for HBV-HCC through the regulation of mRNA expression. These findings provide new insights into the role of nucleotide metabolism-related genetic variation in HBV-HCC progression and may facilitate prognostic assessment, pending replication in independent cohorts.

## Simple Summary

Liver cancer is the third leading cause of cancer death worldwide, with over half of global cases occurring in China. The most common type, hepatocellular carcinoma, is often linked to hepatitis B virus and has a poor prognosis. The aim of our study was to investigate whether potentially functional variants in genes that control nucleotide metabolism—a process that fuels cancer cell growth—affect survival in these patients. We identified two specific genetic variations (in the *DCTD* and *ENTPD2* genes) that were linked to poorer survival. Our findings may help guide personalized treatment and improve patient prognosis.

## 1. Introduction

Liver cancer is the sixth-most commonly diagnosed cancer and the third leading cause of cancer-related mortality worldwide, posing a particularly heavy burden in China [1]. This global burden shows substantial geographical variation, with China accounting for over half of the global mortality in 2022 [2]. Hepatocellular carcinoma (HCC) is the most prevalent histological subtype, accounting for approximately 80% of all primary liver cancer cases [3]. Hepatitis B virus (HBV) is the leading etiological factor globally, responsible for roughly 40% of HCC cases worldwide and up to 84.4% in China [4]. Despite therapeutic advances, the prognosis for patients with HCC remains poor, with a five-year survival rate below 20% [5]. The overall survival (OS) of HCC patients is influenced by multiple clinical factors, including tumor stage, tumor size, histological features, differentiation grade, and vascular invasion [6]. However, substantial heterogeneity in clinical outcomes is common even among patients at the same disease stage [7], suggesting that host genetic factors may play a crucial role in determining HCC survival.

Metabolic reprogramming is a well-established hallmark of cancers, including HCC [8]. Nucleotide metabolism, in particular, supports rapid tumor proliferation by supplying essential precursors for DNA and RNA synthesis, facilitating energy transfer, and regulating signaling processes [9]. Dysregulation of this pathway not only accelerates tumor growth but also promotes chemoresistance and immune evasion [10,11]. Accumulating evidence indicates that genetic variants in nucleotide metabolism-related genes can modulate gene expression and enzyme activity, ultimately influencing prognosis in cancer patients [12]. In the context of HBV-related HCC, this link is particularly compelling, as HBV infection directly reprograms host metabolism to upregulate nucleotide synthesis [13], and the viral HBx protein induces DNA damage that disrupts nucleic acid metabolism [14].

The metabolic landscape of HCC is notably heterogeneous [15]. Aberrant nucleotide metabolism has been consistently implicated in various liver malignancies and contributes significantly to tumor progression [16]. Reprogramming of this pathway has been shown to accelerate HCC cell proliferation by fueling DNA and RNA synthesis [17]. For instance, acetylation of PRPS (phosphoribosyl pyrophosphate synthetase) by nuclear-exported CLOCK (Circadian Locomotor Output Cycles Kaput) enhances de novo nucleotide synthesis and potentially contributes to HCC progression [18]. Similarly, silencing of DUT (deoxyuridine triphosphatase)—a nucleotide metabolic enzyme overexpressed in approximately 42% of HCC tumors—induces cell cycle arrest, promotes spontaneous DNA damage, suppresses proliferation, and reduces sorafenib resistance [19]. A recent bioinformatics analysis of TCGA-HCC data identified eleven nucleotide metabolism-related genes (*UCK2*, *DTYMK*, *CAD*, *RRM2*, *NUDT1*, *TYMS*, *TXNRD1*, *TK1*, *NME1*, *ENTPD2*, and *XDH*) significantly associated with patient prognosis; a prognostic model based on six of these genes (selected by LASSO-Cox regression) demonstrated modest but potentially clinically informative predictive performance [20]. Although the incremental improvement in area under the curve (AUC) was relatively modest, these findings indicate that nucleotide metabolism-related gene expression signatures can provide prognostic information at the transcriptional level in tumor tissues.

Single-nucleotide polymorphisms (SNPs), which represent one of the most common and stable forms of genetic variation [21,22], can influence gene expression and have emerged as potential indicators for predicting cancer susceptibility and survival, including those in HCC [23,24,25]. Candidate gene studies have increasingly demonstrated associations between specific SNPs and HCC survival outcomes [26,27,28], underscoring their potential as survival indicators. Mechanistically, these SNPs may affect gene expression, enzymatic activity, or substrate flux within key metabolic pathways. However, while recent studies have established prognostic models based on nucleotide metabolism-related gene expression in HCC tumor tissues [18], whether germline genetic variants in this pathway also influence HBV-HCC survival remains unexplored. Therefore, we conducted a comprehensive analysis of nucleotide metabolism-related genetic variants in a retrospective cohort of patients with HBV-related HCC to evaluate their clinical utility as potential prognostic indicators that may assist in refining risk assessment.

## 2. Materials and Methods

### 2.1. Study Populations

We conducted this retrospective cohort study, which included 866 patients with histologically confirmed HBV-HCC, all of whom underwent surgical resection at Guangxi Medical University Cancer Hospital from June 2007 to December 2017 [28,29]. The inclusion and exclusion criteria have been previously described in detail [29]. Briefly, patients with pathologically confirmed HCC who were seropositive for HBsAg and underwent curative hepatectomy were included, while those with anti-HCV seropositivity, distant metastasis, severe comorbidities, or prior neoadjuvant therapy were excluded. Peripheral blood samples (5 mL) were collected preoperatively for genomic DNA extraction; the genotyped SNPs therefore represent host germline polymorphisms rather than somatic mutations. Standardized demographic and clinical data were collected, including age, sex, smoking status, alcohol consumption, alpha-fetoprotein (AFP) levels, cirrhosis status, cancer embolus, and Barcelona Clinic Liver Cancer (BCLC) stage. OS was calculated from the date of surgical resection to the date of death or last follow-up. The follow-up schedule involved assessments at 3-month intervals for the first 2 years, transitioning to 6-month intervals thereafter, with the final follow-up completed by 31 March 2020. Ethical approval for this study was granted by the Institutional Review Board of Guangxi Medical University Cancer Hospital (approval number: KY20251033), with all patients having signed written informed consent forms.

### 2.2. Genotyping, Gene Selection and SNPs

Genotyping was performed with the Illumina Infinium^®^ Global Screening Assay (Shanghai, China) [30]. Candidate genes involved in nucleotide metabolism were systematically identified through the MSigDB database https://www.gsea-msigdb.org/gsea/msigdb/search.jsp (accessed on 7 June 2026) using the keyword “nucleotides”. After excluding five genes, three of which are located on the X chromosome (*CTPS2*, *HPRT1*, and *PUDP*), 94 candidate genes were selected for further analysis (Table S1). SNPs within these candidate genes, including the 2 kb upstream and downstream flanking regions, were extracted from the 1000 Genomes Project (March 2012 release, Han Chinese in Beijing [CHB] population). Quality control (QC) criteria were applied as follows: (a) a genotyping call rate > 95%; (b) minor allele frequency (MAF) > 5%; and (c) Hardy–Weinberg equilibrium (HWE) with a *p*-value ≥ 10^−6^. After QC filtering, 10,825 SNPs were retained for subsequent analyses.

### 2.3. Expression Quantitative Trait Loci (eQTL) Analysis and Functional Prediction

To identify and prioritize potentially functional SNPs, we performed a multi-step bioinformatics analysis. First, eQTL analysis was conducted using data from 261 normal liver tissues and 800 blood samples from the Genotype-Tissue Expression (GTEx) portal https://www.gtexportal.org (accessed on 7–8 June 2026) [31]. SNPs with a significance level of *p* < 0.001 were retained for further functional annotation. We then employed three complementary tools for in silico functional prediction: RegulomeDB (R-DB) https://regulomedb.org/ (accessed on 8 June 2026), which integrates multi-omics evidence to assign confidence scores from 1 (most likely functional) to 7 (least likely)—with variants scoring ≤ 3a considered of moderate to high regulatory potential; the SNPinfo Web Server https://snpinfo.niehs.nih.gov/ (accessed on 8 June 2026), used to predict effects on transcription factor binding sites (TFBS), miRNA targeting, and splicing regulation [32]; and HaploReg v4.2 https://pubs.broadinstitute.org/mammals/haploreg/haploreg.php (accessed on 8 June 2026) for additional regulatory motif and conservation annotation [33]. Additionally, we used 3DSNP2.0 https://omic.tech/3dsnpv2/ (accessed on 8 June 2026) to further predict the function of candidate genes [34]. Tagging SNPs were identified via LD analysis (r^2^ ≥ 0.8) using HaploView 4.2 [35]. Finally, to examine broader genomic alterations in candidate genes, we analyzed mutation rates and copy number alterations using the cBioPortal for Cancer Genomics database http://www.cbioportal.org (accessed on 8 June 2026) [36].

### 2.4. Differential Gene Expression Analysis

To evaluate differential gene expression, we first analyzed RNA sequencing data from tumor and paired normal tissues from an additional cohort of 103 HCC patients who underwent hepatectomy at Guangxi Medical University Cancer Hospital. We then extended the analysis using the UALCAN database https://ualcan.path.uab.edu/ (accessed on 4 July 2026) [37] to examine expression differences between HCC and normal tissues. Additionally, we employed UALCAN to assess the associations between mRNA expression levels and OS in HCC.

To explore the potential link between gene expression and the immune microenvironment, we performed immune cell infiltration analysis using publicly available liver hepatocellular carcinoma (LIHC) transcriptomic data. Regulatory T-cell (Treg) infiltration scores were estimated using multiple algorithms, including CIBERSORT, CIBERSORT-ABS, and QUANTISEQ. Spearman’s rank correlation test was used to assess the correlation between gene expression and Treg infiltration.

### 2.5. Dual-Luciferase Reporter Assay

To elucidate the functional effect of candidate SNPs on gene expression regulation, we performed dual-luciferase reporter assays. Briefly, genomic fragments (500 bp) encompassing *ENTPD2* rs3763662 (Table S2) were cloned into the pGL3-basic vector (Promega) to generate three constructs per SNP: an empty vector control, a major allele, and a minor allele variant. HEK-293T cells (Chinese Academy of Sciences, Shanghai, China) and SNU449 cells (Guangzhou Cellcook Biotech, Guangzhou, China) cultured in DMEM and RPMI 1640, respectively, both supplemented with 10% FBS and 1% penicillin–streptomycin (37 °C, 5% CO_2_), were seeded in 96-well plates (1 × 10^4^ cells/well for HEK-293T and 1.5 × 10^4^ cells/well for SNU449) and transfected after 24 h with 100 ng of each plasmid construct (empty vector, major allele, or minor allele) using Effectene Transfection Reagent (QIAGEN, Hilden, Germany). For normalization, 10 ng of the Renilla luciferase pRL-TK plasmid was co-transfected. Luciferase activity was measured 24 h (HEK-293T cells) and 36 h (SNU449 cells) post-transfection using the Dual-Luciferase Reporter Assay System (Promega, Madison, WI, USA). Each condition was independently replicated three times to ensure reproducibility. All experiments were performed with mycoplasma-free cells. Both HEK-293T cells and SNU449 cells were authenticated by STR profiling within the previous three years.

### 2.6. Statistical Analysis

We employed a multivariable Cox proportional hazards regression model, adjusting for age, sex, smoking status, drinking status, AFP level, cirrhosis, cancer embolus, and BCLC stage, to evaluate the associations between SNPs and OS in HBV-HCC patients. For all single-SNP association analyses, the additive genetic model (coding genotypes as 0, 1, or 2 according to the number of effect alleles) was pre-specified as the primary analysis to maximize statistical power and ensure comparability with previous studies. To address the multiple comparison issue, we applied the Bayesian false discovery probability (BFDP) and the false-positive report probability (FPRP) for multiple testing correction, with cutoff values of 0.80 and 0.20, respectively [38,39]. For both measures, the prior probability of a true association was set at π_1_ = 0.01. For FPRP, the effect size for power calculation was specified as OR = 1.5; for BFDP, the prior variance was specified as (log(2)/1.96)^2^, corresponding to a prior OR of 2.0. To construct the final multivariate Cox models, we performed a bidirectional stepwise Cox regression procedure using the Akaike Information Criterion (AIC) for variable selection; the stepAIC function was applied with direction = “both”, where variables were iteratively added and removed to minimize the AIC until the model with the lowest AIC was achieved. The candidate variables included the prioritized SNPs and all clinical covariates listed above.

For the identified SNPs, we further performed dominant model (risk allele carriers vs. non-carriers) analyses as exploratory analyses to characterize their genetic effects more comprehensively. The cumulative effects of the risk alleles of the identified SNPs were subsequently assessed by multivariable Cox regression and Kaplan–Meier (KM) survival curves. Stratified analyses were further performed as exploratory analyses to detect multiplicative interactions between identified SNPs and clinical/demographic variables. The proportional hazards (PH) assumption for the final Cox models was assessed using the Schoenfeld residual-based test; a non-significant global test (*p* > 0.05) indicated that the PH assumption was satisfied.

To assess potential bias in the identification of significant variants, we performed bootstrap resampling with 1000 replicates and calculated the hazard ratios (HRs) and corresponding 95% confidence intervals (CIs) as exploratory analyses. The normality of the bootstrap HR distributions was evaluated using the Shapiro–Wilk test; an original HR falling within the bootstrap 95% CI indicated robustness against sampling variability. The survival prediction performance of the identified SNPs was assessed using time-dependent receiver operating characteristic (ROC) curve analysis, generated via inverse probability of censoring weighting to handle censoring, with AUC estimates and 95% CIs derived from the iid (independent and identically distributed) representation method [40]. The significance of AUC differences between models with and without the genetic risk score (GRS) was assessed using a non-parametric test [40]. The GRS was constructed as a simple unweighted score by counting the number of unfavorable genotypes, yielding a score from 0 to 2, corresponding to the NUG (number of unfavorable genotypes). Regional plots were generated using LocusZoom http://locuszoom.sph.umich.edu (accessed on 8 June 2026) [41]. All statistical analyses were performed using R software (versions 3.1.3 and 4.1.3). The R packages included “MASS”, “survminer”, “survival”, “gap”, “GenABLE” and “timeROC”. For all statistical analyses, a two-sided *p* < 0.05 was considered statistically significant.

## 3. Results

### 3.1. Characteristics of the Study Population

This study enrolled a total of 866 Chinese patients with HBV-related HCC who underwent curative hepatectomy, with a median follow-up duration of 39.05 months. The cohort was predominantly male (87.4%, *n* = 760), with a median age at diagnosis of 47 years. Multivariable Cox regression analysis identified age, AFP level, cancer embolus, and BCLC stage as independent prognostic factors for OS. Specifically, advanced BCLC stages (B/C) were associated with nearly twice the risk of death compared to early stages (0/A) (HR = 1.98, 95% CI: 1.56–2.52, *p* < 0.001). Elevated AFP (HR = 1.29, 95% CI: 1.05–1.57, *p* = 0.015) and the presence of cancer embolus (HR = 1.74, 95% CI: 1.38–2.21, *p* < 0.001) were also significantly associated with poorer OS (Table S3). Interestingly, older age at diagnosis was associated with a reduced risk of mortality (HR = 0.81, 95% CI: 0.66–0.99, *p* = 0.036), which may reflect a survivor effect or differences in tumor biology among age groups.

### 3.2. Associations Between Candidate SNPs in Nucleotide Metabolism Pathway Genes and HBV-HCC Survival

**Figure 1:**
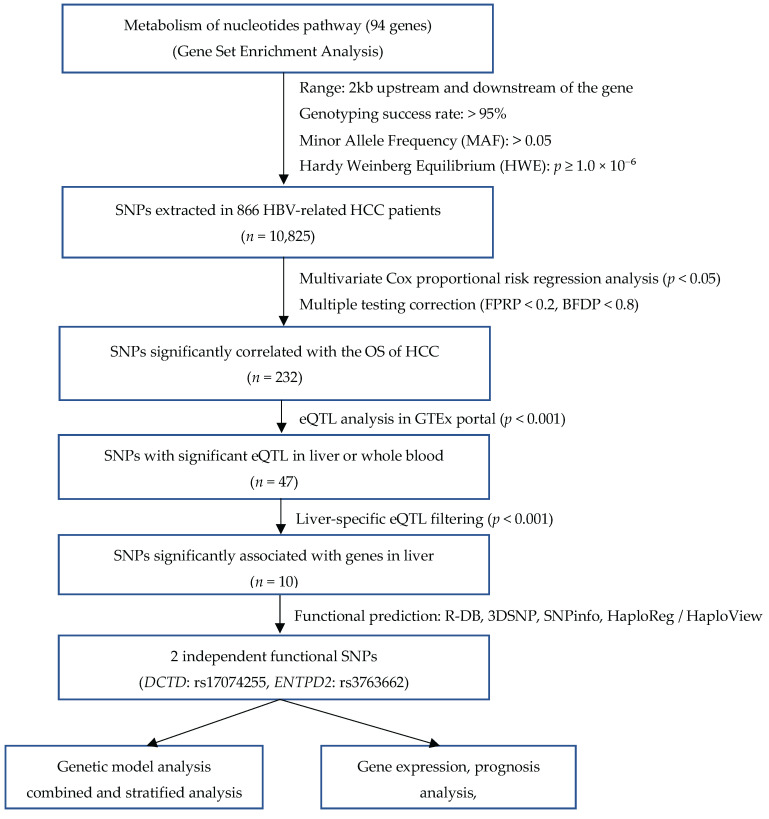
Flowchart of the present study design.

As illustrated in the workflow (Figure 1), 94 nucleotide metabolism pathway genes were selected as candidate genes (Table S1). After QC, 10,825 SNPs were retained for analysis. Single-locus analysis identified 232 SNPs significantly associated with OS in HBV-HCC patients (*p* < 0.05, BFDP  <  0.8, FPRP < 0.2). To prioritize functionally relevant variants, we performed eQTL analysis using GTEx data and identified 47 SNPs with significant eQTL in liver tissue or whole blood (*p* < 0.001) (Table S4), of which 10 exhibited liver-specific eQTL signals and were prioritized for further evaluation (Table 1). Functional annotation using an R-DB score ≤ 3a and SNPinfo (TFBS) identified two candidate SNPs: one in *DCTD* (rs17074255, R-DB score = 3a) and one in *ENTPD2* (rs3763662, R-DB score = 2a) (Table 1). As each gene contained only one qualifying SNP, both were selected for further validation. Multivariable Cox regression confirmed both SNPs (rs17074255 G>A: HR = 1.22, 95% CI: 1.06–1.40, *p* = 0.005; rs3763662 G>A: HR = 1.18, 95% CI: 1.03–1.34, *p* = 0.015) as independent prognostic markers (Table 2). Regional association plots further illustrated the genomic context of these loci (Figure S1).

**Figure 2:**
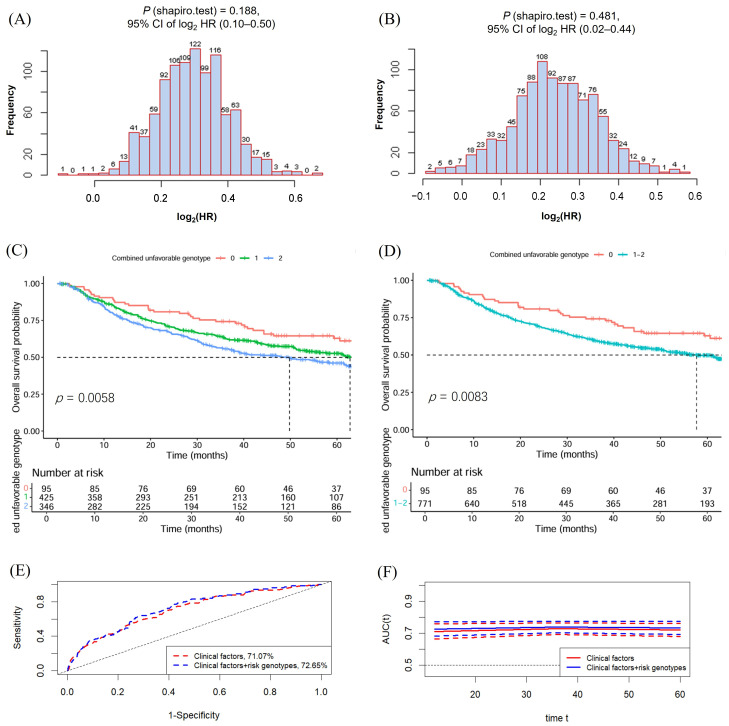
Two identified significant SNPs in nucleotide metabolism pathway genes predict the overall survival of HBV-HCC patients. (**A**) Distribution histogram of 1000 HR estimates for rs17074255 obtained from 1000 bootstrap resamples. (**B**) Distribution histogram of 1000 HR estimates for rs3763662 obtained from 1000 bootstrap resampling. Kaplan–Meier survival curves for overall survival in the HBV-HCC dataset for (**C**) the combined protective alleles and (**D**) dichotomized groups of the NUGs. (**E**) One-year HCC overall survival prediction by ROC curve (*p* = 0.052). The red dashed line represents the clinical factors model, and the blue dashed line represents the clinical factors combined with the risk genotypes model; (**F**) Time-dependent AUC estimation for covariables plus protective alleles (*p* = 0.091). Solid lines represent the AUC estimates, and dashed lines indicate the 95% confidence intervals. In panels (**C**,**D**), unfavorable genotypes were *DCTD* rs17074255 GA + AA and *ENTPD2* rs3763662 GA + AA. Abbreviations: HR, hazard ratio; SNPs, single-nucleotide polymorphisms; NUG, number of unfavorable genotypes; ROC, receiver operating characteristic curve; AUC, area under the curve.

To confirm that the observed significance of *DCTD* rs17074255 G>A and *ENTPD2* rs3763662 G>A was unlikely to be attributable to random error, we implemented bootstrapping with 1000 replicates and derived the corresponding HR estimates. As shown in Figure 2A,B, the bootstrapped HRs were normally distributed (*P*_shapiro.test_ > 0.05), and the original log_2_HR estimates (0.32 for *DCTD* rs17074255 G>A and 0.24 for *ENTPD2* rs3763662 G>A) both fell within the 95% CI of the bootstrap distributions (0.10–0.50 and 0.02–0.44, respectively). These results suggest the reliability and stability of our findings.

### 3.3. Combined and Stratified Analyses of Independent SNPs Associated with HBV-HCC Survival and Their Interactions with Risk Factors

As shown in Table 3, both the A allele of *DCTD* rs17074255 and *ENTPD2* rs3763662 were associated with poorer survival in HBV-HCC patients (*p_trend_* = 0.005 and 0.013, respectively). Under a dominant genetic model, patients carrying both risk genotypes (GA/AA) showed significantly poorer survival compared with those with the GG reference genotype (HR = 1.32, 95% CI: 1.07–1.63, *p* = 0.010 for rs17074255; HR = 1.28, 95% CI: 1.05–1.57, *p* = 0.017 for rs3763662). To evaluate the cumulative effect of these variants, a simple unweighted GRS was constructed by summing the number of risk/unfavorable alleles (the A allele of *DCTD* rs17074255 and the A allele of *ENTPD2* rs3763662), yielding a score from 0 to 2. A dose–response relationship was observed between higher GRS and poorer OS after adjustment for clinical covariates (*p-_trend_* < 0.001). Patients were categorized into low-risk (GRS = 0) and high-risk (GRS = 1–2) groups. Those in the high-risk group had significantly poorer survival (HR = 1.67, 95% CI: 1.19–2.34, *p* = 0.003), with clear separation in the KM curves (Figure 2C,D).

The predictive performance of the SNPs was assessed by comparing time-dependent ROC curves. Incorporation of the GRS improved the AUC for 1-year survival prediction from 71.07% to 72.65% (*p* = 0.052, Figure 2E). However, no significant improvement was observed for 3-year survival (AUC: 72.72% to 73.78%, *p* = 0.091) or 5-year survival (AUC: 72.04% to 73.30%, *p* = 0.102) (Figure S2A,B). The time-dependent AUCs are shown in Figure 2F.

Stratified analyses indicated that patients with 1–2 risk alleles consistently exhibited poorer survival across most clinical subgroups. Exceptions included younger patients (≤47 years), females, smokers, non-drinkers, those with AFP > 400 ng/mL, cirrhosis, absence of embolus, and BCLC stage 0/A (Table S5). No significant multiplicative interactions were observed between the GRS and any clinical variables, including age, gender, or smoking status (Table S5).

### 3.4. Bioinformatic Functional Annotation of Prognostic SNPs

Functional annotation of the two independent prognostic SNPs was performed using HaploReg v4.2. The results indicated that the *DCTD* rs17074255 G>A variant is located within a functional regulatory region characterized by promoter histone marks, enhancer histone marks, DNase hypersensitivity, protein binding and transcription factor-binding motifs. Similarly, the *ENTPD2* rs3763662 G>A variant resides in a region enriched for promoter and enhancer histone marks, DNase hypersensitivity, and transcription factor-binding motifs, suggesting its potential role in gene regulation (Table 1).

In addition, we explored the correlation between *DCTD*/*ENTPD2* mRNA expression and Treg infiltration in HCC using publicly available LIHC immune deconvolution data. As shown in Figure S6, the expression of both genes was positively correlated with Treg infiltration across multiple algorithms (CIBERSORT, CIBERSORT-ABS, and QUANTISEQ), suggesting that higher expression of these nucleotide metabolism-related genes may be associated with a more immunosuppressive tumor microenvironment.

### 3.5. eQTL Analysis and Dual-Luciferase Reporter Assay

**Figure 3:**
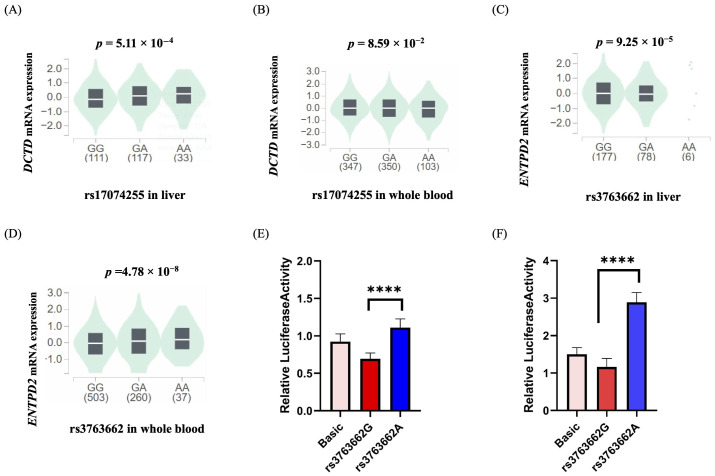
eQTL analysis of *DCTD* rs17074255 and *ENTPD2* rs3763662 and the dual-luciferase reporter assay. The correlation between rs17074255 genotypes and *DCTD* mRNA expression in (**A**) normal liver tissue (n = 261, *p* = 5.11 × 10^−4^) and (**B**) whole blood (n = 800, *p* = 8.59 × 10^−2^). The correlation between rs3763662 genotypes and *ENTPD2* mRNA expression in (**C**) normal liver tissue (n = 261, *p* = 9.25 × 10^−5^) and (**D**) whole blood (n = 800, *p* = 4.78 × 10^−8^); statistical significance was assessed using linear regression, with exact *p* values shown. (**E**,**F**) Normalized reporter gene activity from the constructed fragments of *ENTPD2* rs3763662 G>A in the HEK-293T and SNU449 cell lines; data are presented as mean ± SD from three independent biological replicates, each with three technical replicates; statistical significance was assessed using the two-tailed Student’s *t* test. Abbreviations: eQTL, expression quantitative trait. **** *p* < 0.0001.

We performed an eQTL analysis using data from 261 normal liver tissues and 800 whole-blood samples derived from the GTEx project. The analysis revealed a significant association between the rs17074255 A allele and elevated *DCTD* mRNA expression in normal liver tissues (*p* = 5.11 × 10^−3^, Figure 3A), but no significant association was observed in whole-blood samples (*p* = 8.59 × 10^−2^, Figure 3B). Additionally, the rs3763662 A allele showed a significant association with elevated *ENTPD2* mRNA expression in normal liver tissues (*p* = 9.25 × 10^−5^, Figure 3C) and in whole-blood samples (*p* = 4.78 × 10^−8^, Figure 3D).

To further validate the functional effect of rs3763662, we conducted luciferase reporter assays in HEK-293T cells (Figure 3E) and SNU449 cells (Figure 3F). GTEx-based eQTL analysis suggested that the rs3763662 A allele was associated with increased *ENTPD2* expression in normal liver tissues and whole blood. Consistently, luciferase reporter assays also demonstrated increased transcriptional activity of the A allele in HEK-293T and SNU449 cells. The concordant results between the eQTL analysis and the in vitro reporter assay provide supportive evidence that rs3763662 functions as a regulatory variant affecting gene expression. These findings strengthen the biological plausibility of rs3763662, while additional mechanistic studies are still needed to further elucidate its context-dependent regulatory mechanisms.

### 3.6. Differential mRNA Expression Analysis and Survival of HCC

**Figure 4:**
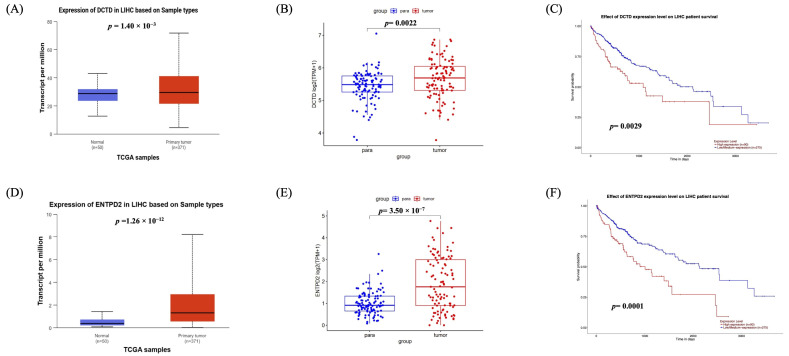
Differential mRNA expression analysis and overall survival analysis of *DCTD* and *ENTPD2* in the UALCAN database and our samples. (**A**,**D**): Higher *DCTD* and *ENTPD2* mRNA expression levels in LIHC tissues compared to the normal tissues in the UALCAN database; statistical significance was assessed using the unpaired Student’s *t*-test. (**B**,**E**): Higher *DCTD* and *ENTPD2* mRNA expression levels in HCC tissues compared to the normal tissues in our 103 paired tumor and liver tissue samples; statistical significance was assessed using the paired Student’s *t*-test. (**C**,**F**): Higher expression levels of *DCTD* and *ENTPD2* were correlated with poorer survival; statistical significance was assessed using the log-rank test, with exact *p* values shown. Abbreviations: LIHC, liver hepatocellular carcinoma.

As shown in Figure 4A, using the UALCAN database, the mRNA expression levels of *DCTD* were significantly increased in tumor tissues of LIHC compared with normal tissues (*p* = 1.40 × 10^−3^). Similarly, the mRNA expression levels of *ENTPD2* were significantly elevated in tumor tissues (*p* = 1.26 × 10^−12^) (Figure 4D). A similar pattern was observed in our collection of 103 paired tissue samples (*DCTD*: *p* = 0.002; *ENTPD2*: *p* = 3.5 × 10^−7^, respectively) (Figure 4B,E). Most importantly, survival analysis using the UALCAN database demonstrated that patients with higher *DCTD* and *ENTPD2* mRNA expression exhibited significantly poorer OS in HCC patients (*p* = 3.00 × 10^−3^ and *p* = 1.30 × 10^−4^, Figure 4C,F), and the same trend was observed in our 103 HCC cases (Figure S3A,B). Consistently, analysis of KM-plot survival data showed that higher *ENTPD2* mRNA expression levels were associated with poor OS in patients with HCC (*p* = 0.0007) (Figure S4A). In contrast, increased *DCTD* mRNA expression was associated with better OS without statistical significance (*p* = 0.11), although a trend toward improved survival was observed (Figure S4B). Interestingly, however, increased *DCTD* mRNA expression was associated with poorer OS in women’s HCC (*p* = 0.045) (Figure S4C).

### 3.7. Mutation Analyses

Finally, we investigated the mutation status of *DCTD* and *ENTPD2* in HCC tissues using the cBioPortal for Cancer Genomics database. As shown in Figure S5, *DCTD* exhibited a relatively low somatic mutation rate in HCC (0.27% in the TCGA GDC cohort, 0.2% in the CLCA cohort, 0.27% in the TCGA Firehose Legacy study, 0.27% in the TCGA PanCancer Atlas study). Similarly, *ENTPD2* also demonstrated a relatively low somatic mutation rate in HCC (1.75% in the MERiC/Basel, 0.61% in the CLCA cohort, 0.55% in the TCGA PanCancer Atlas study, 0.55% in the TCGA GDC cohort, and 0.27% in the TCGA Firehose Legacy study). These low somatic mutation frequencies suggest that transcriptional dysregulation, rather than recurrent coding mutations, may contribute more substantially to the functional involvement of *DCTD* and *ENTPD2* in HCC.

## 4. Discussion

This study systematically evaluates nucleotide metabolism pathway-related genetic variants in relation to survival outcomes in HBV-related HCC. We identified two potentially functional SNPs—*DCTD* rs17074255 G>A and *ENTPD2* rs3763662 G>A—for which the A allele was significantly associated with OS in HBV-HCC patients. A combined GRS further revealed a dose-dependent relationship between the number of risk alleles and poorer survival.

The eQTL analysis demonstrated that the rs3763662 A allele was associated with higher *ENTPD2* mRNA expression in both normal liver and whole blood, a finding further validated by dual-luciferase reporter assays in HEK-293T and SNU449 cell lines. The rs17074255 A allele was significantly associated with higher *DCTD* expression in normal liver tissue, while its effect in whole blood was not statistically significant, potentially due to a weaker effect in blood than in tissue. Consistent with a potential oncogenic role, both *DCTD* and *ENTPD2* mRNA levels were significantly higher in HCC tissues than in adjacent normal samples, as evidenced by the UALCAN database and validation in our 103 paired clinical specimens. Moreover, higher expression of both genes was associated with poorer survival in HCC patients based on the UALCAN cohort.

HBV-related hepatocarcinogenesis is characterized by chronic inflammation, sustained hepatocyte regeneration, viral DNA replication, and profound metabolic reprogramming. These processes impose substantial demands on nucleotide biosynthesis and DNA repair pathways. Given that HBV replication itself depends on host nucleotide pools and contributes to chronic hepatic injury and regeneration, dysregulation of nucleotide metabolism may exert particularly important effects in HBV-associated hepatocarcinogenesis [13]. Therefore, genetic variants affecting nucleotide metabolism may exert particularly pronounced biological consequences in HBV-HCC by influencing proliferative capacity, genomic stability, and adaptation to the chronically inflamed hepatic microenvironment.

*DCTD* (dCMP deaminase), located on chromosome 4q35.1, encodes a key enzyme in nucleotide metabolism that catalyzes the conversion of deoxycytidylate (dCMP) to deoxyuridylate (dUMP), a reaction essential for DNA synthesis and repair [42,43]. Aberrant expression of *DCTD* may disrupt nucleotide pool balance and compromise genetic stability, thereby supporting rapid tumor proliferation. As such, DCTD may function as a key metabolic enzyme supporting nucleotide biosynthesis during cancer progression by meeting the heightened demand for nucleotide precursors. Studies across multiple cancers support an oncogenic role for *DCTD*. In glioma, analyses of CGGA and TCGA data revealed that elevated *DCTD* expression correlates with advanced tumor grade, reduced promoter methylation, and poorer patient survival. Functional enrichment analyses further suggested that high *DCTD* expression was associated with enhanced cell proliferation, epithelial–mesenchymal transition, inflammatory responses, and cell adhesion—processes closely linked to tumor invasion and immune evasion. Notably, *DCTD* exhibited high sensitivity and specificity (>70%) in predicting 3- and 5-year survival in glioma patients, highlighting its potential as a prognostic biomarker [44]. Additionally, DCTD deficiency was shown to enhance decitabine cytotoxicity via nucleotide metabolism disruption [45], whereas its low expression promoted gemcitabine resistance in triple-negative breast cancer by suppressing apoptosis and sustaining proliferation [46].

In line with these findings, our study demonstrated that the *DCTD* rs17074255 G>A was associated with increased *DCTD* mRNA expression in normal liver tissue and with poorer OS in HBV-HCC patients. We also observed significant upregulation of *DCTD* in HCC tissues compared to normal liver samples. Analysis of the UALCAN database further confirmed that high *DCTD* expression predicts reduced OS in HCC. Collectively, these results suggest that DCTD may play a cancer-promoting role in HCC, likely by supporting nucleotide metabolism to promote tumor growth and progression. Nevertheless, several considerations warrant cautious interpretation. Unlike *ENTPD2* rs3763662, no direct reporter assay was performed for *DCTD* rs17074255, and its eQTL association with *DCTD* mRNA expression was significant in normal liver tissue but not in whole blood, suggesting a tissue-specific regulatory effect that requires further validation. Additionally, the association between higher *DCTD* mRNA expression and poorer survival, while significant in the UALCAN dataset, did not reach significance in the KM-plot analysis—a discrepancy that may reflect differences in data source composition or analytical approaches, such as cutoff selection and stratification methods. Despite these uncertainties, the consistent elevation of *DCTD* mRNA expression in tumor tissues (observed in both UALCAN and our 103 paired samples) supports its potential involvement in HCC. Overall, *DCTD* may contribute to HCC progression, but its utility as a prognostic indicator requires further mechanistic and validation studies.

*ENTPD2* (Ectonucleoside Triphosphate Diphosphohydrolase 2), located on chromosome 9q34.3, encodes an integral membrane protein involved in hydrolase activity, nucleoside diphosphate phosphatase activity, and nucleotide metabolism. By regulating the balance of extracellular ATP and adenosine, ENTPD2 critically influences tumor immunomodulation. This role was exemplified in colorectal cancer, where ENTPD2-mediated ATP-adenosine metabolism reprogramming suppressed CD8^+^ T-cell function and promoted an immunosuppressive niche, thereby facilitating tumor progression [47]. The immunomodulatory impact of *ENTPD2* extends across multiple malignancies. It was overexpressed in HCC and has been identified as a poor prognostic biomarker [48], and similarly served as an indicator of unfavorable prognosis in gastric cancer, where it constitutes part of a six-gene immune-microenvironment-related prognostic signature (including *CTLA4*, *CLDN6*, *EMB*, *GPR15*, *VWF*, and *AKR1B1*) with strong predictive accuracy for 3- and 5-year OS (AUC = 0.764 and 0.802, respectively) [49]. *ENTPD2* has also been recognized as a tumor immune-related prognostic gene in lung adenocarcinoma [50]. A key mechanism underlying its pro-tumorigenic effect involves the elevation of extracellular 5′-AMP in HCC, which maintains myeloid-derived suppressor cells by preventing their differentiation, thereby possibly contributing to immune evasion [51]. Consistent with these findings, *ENTPD2* is frequently overexpressed across multiple malignancies, where it enhances tumor cell proliferation, migration, and invasion [47,52].

In line with these reports, our study found that *ENTPD2* rs3763662 G>A was associated with both increased mRNA expression in normal liver and reduced OS in HBV-HCC patients. We also observed significant upregulation of *ENTPD2* in HCC tissues compared to normal liver samples, with higher expression correlating with poorer survival. One possible explanation is that the rs3763662 A allele may influence transcription factor binding or modify chromatin architecture in the *ENTPD2* promoter region, thereby increasing its expression. Further mechanistic studies are warranted to elucidate how *ENTPD2* genetic variants influence survival outcomes in HCC.

A notable feature of HBV-related hepatocarcinogenesis is the immune-suppressive microenvironment shaped by chronic viral infection [53]. HBV exposure has been shown to drive the expansion of CCR4^+^ Tregs, a highly suppressive Treg subset that is significantly correlated with serum HBV viral load and contributes to immune evasion in HBV-HCC [54]. In our exploratory analysis, we observed positive correlations between *DCTD/ENTPD2* mRNA expression and Treg infiltration in LIHC transcriptomic data. Given that nucleotide metabolism provides essential precursors for DNA synthesis and supports the proliferation and functional maintenance of both tumor cells and immune cells, it is plausible that genetic variants affecting nucleotide metabolic flux—such as those identified in *DCTD* and *ENTPD2*—might influence the equilibrium between effector immune responses and Treg-mediated immunosuppression. This is particularly relevant for *ENTPD2*, which encodes an ectonucleotidase that regulates the balance of extracellular ATP and adenosine, a key axis in tumor immunomodulation [47]. However, the direct mechanistic link between the two SNPs identified in this study and Treg-mediated immunosuppression remains hypothetical and requires dedicated immunological investigation. Future studies integrating genetic, transcriptomic, and immune profiling data are warranted to explore this potential connection.

While our study provides valuable insights into the genetic determinants of survival in HBV-HCC, several limitations should be acknowledged. First, although the single-center cohort of Chinese patients ensured consistent surgical protocols and follow-up, minimizing clinical heterogeneity, it inevitably limits the extrapolation of our findings to other ethnicities or HBV-endemic regions, such as Africa, where distinct HBV genotypes and environmental cofactors may modulate the effects of host genetic variations [55,56]. Although BFDP and FPRP were applied to reduce false-positive findings, the residual risk of type I error cannot be completely excluded given the large number of variants analyzed. Future studies involving multicenter, large-scale, and multi-ethnic prospective cohorts (particularly in African and European populations with different HBV genotypes) are warranted to validate these results. Second, although core clinical variables were available, detailed information on tumor size, tumor number, pathological differentiation, microvascular invasion, antiviral therapy, postoperative recurrence, and subsequent treatments such as TACE (transcatheter arterial chemoembolization), targeted therapy, immunotherapy, or repeat surgery was largely absent, preventing comprehensive adjustment for these potential confounders. In addition, because genotype and tumor transcriptome data were not directly paired in the same patients, the causal relationship between the identified SNPs and tumor-specific gene expression requires further validation. Furthermore, while our luciferase reporter assays demonstrated allele-specific regulatory activity, the specific transcription factors and chromatin architecture mediating these effects were not identified, and the precise molecular mechanisms require further investigation.

## 5. Conclusions

Our study identified two potentially functional SNPs—*DCTD* rs17074255 G>A and *ENTPD2* rs3763662 G>A—that were significantly associated with OS in HBV-HCC patients, likely through the modulation of their respective gene expression levels. These results underscore the importance of nucleotide metabolism pathway genes in HCC progression and suggest that genetic variants in this pathway may serve as potential prognostic indicators for HBV-HCC, pending further validation in independent cohorts and mechanistic investigations.

## Figures and Tables

**Table 1:** Associations of 10 significant SNPs with overall survival in HBV-HCC patients and the corresponding bioinformatic functional annotations.

SNP	Chr	Gene	Allele ^a^	MAF	HR (95% CI)	*p* ^b^	BFDP	FPRP	eQTL	R-DB ^c^	3DSNP ^d^	SNPinfo ^e^	Haploreg V4.2 ^f^
									*p-Liver*	Ranking	SCORE		Motifs Changed
**rs17074255**	**4**	***DCTD***	**G>A**	**0.41**	**1.21** **(1.06–1.39)**	**0.006**	**0.583**	**0.060**	5.10 × 10^−4^	**3a**	**139.49**	**TFBS**	**HP1-site-factor, Pax-4, RFX5**
rs9411300	9	*ENTPD2*	T > A	0.41	1.17 (1.02–1.34)	0.021	0.797	0.173	8.50 × 10^−4^	5	51.84	TFBS	NRSF, SETDB1
rs3814504	9	*ENTPD2*	G > C	0.41	1.18 (1.03–1.35)	0.016	0.742	0.125	6.50 × 10^−4^	4	31.35	TFBS	11 altered motifs
rs17853460	9	*ENTPD2*	A > G	0.41	1.18 (1.03–1.35)	0.015	0.742	0.125	3.10 × 10^−4^	4	29.64	--	CTCF, Rad21, SRF
rs2292925	9	*ENTPD2*	G>A	0.41	1.19 (1.04–1.36)	0.012	0.672	0.088	1.10 × 10^−4^	4	19.65	--	7 altered motifs
rs4880084	9	*ENTPD2*	C > T	0.41	1.18 (1.03–1.35)	0.016	0.742	0.125	6.50 × 10^−5^	3a	20.32	--	6 altered motifs
rs9411242	9	*ENTPD2*	G>A	0.41	1.18 (1.03–1.35)	0.015	0.742	0.125	6.50 × 10^−5^	4	72.83	TFBS	Egr-1, GLI, Zic
**rs3763662**	**9**	***ENTPD2***	**G>A**	**0.41**	**1.19** **(1.04–1.36)**	**0.013**	**0.672**	**0.088**	9.20 × 10^−5^	**2a**	**88.7**	**TFBS**	**AP-1, Egr-1, Sox**
rs9953424	18	*ENOSF1*	C > T	0.28	0.80 (0.68–0.94)	0.007	0.548	0.057	2.00 × 10^−8^	5	4.15	--	Egr-1, SP1
rs9948583	18	*ENOSF1*	T > C	0.29	0.83 (0.71–0.97)	0.022	0.751	0.147	2.20 × 10^−8^	1f	5.85	--	INSM1, Myc, UF1H3BETA

SNPs in bold indicate the two independent SNPs that were finally prioritized for further evaluation. Abbreviations: SNPs, single-nucleotide polymorphisms; HBV, hepatitis B virus; HCC, hepatocellular carcinoma; MAF, Minor Allele Frequency; HR, hazard ratio; 95% CI, 95% confidence interval; BFDP, Bayesian false discovery probability; eQTL, expression quantitative trait locus; TFBS, transcription factor binding site. ^a^ Referring allele/effect allele. ^b^ Multivariate Cox proportional hazards regression analysis was adjusted for age, sex, smoking status, drinking status, AFP level, cirrhosis, embolus, and BCLC stage. ^c^ http://www.regulomedb.org/index. ^d^ https://omic.tech/3dsnpv2. ^e^ http://snpinfo.niehs.nih.gov/snpinfo/snpfunc.html. ^f^ https://pubs.broadinstitute.org/mammals/haploreg/haploreg.php.

**Table 2:** Two independent SNPs identified by Cox proportional hazards regression analysis with adjustment for other covariates in 866 HBV-HCC patients.

Characteristics	Category	No. of Patients	HR (95% CI)	*p* ^a^
Age (year)	≤47	434	1.00	
	>47	432	0.80 (0.66–0.97)	0.026
AFP level (ng/mL)	≤400	522	1.00	
	>400	344	1.25 (1.02–1.53)	0.029
Embolus	No	636	1.00	
	Yes	230	1.73 (1.37–2.20)	<0.001
BCLC stage	0/A	427	1.00	
	B/C	439	2.03 (1.60–2.58)	<0.001
*DCTD* rs17074255 G>A	GG/GA/AA	300/418/148	1.22 (1.06–1.40)	0.005
*ENTPD2* rs3763662 G>A	GG/GA/AA	315/396/155	1.18 (1.03–1.34)	0.015

Abbreviations: HR: hazard ratio; 95% CI: 95% confidence interval; AFP: alpha-fetoprotein; BCLC: Barcelona Clinic Liver Cancer. ^a^ Stepwise Cox regression analysis was performed with age, sex, smoking status, drinking status, AFP level, cirrhosis, cancer embolus, BCLC stage and the two prioritized SNPs as candidates.

**Table 3:** Associations between the two identified SNPs and overall survival of HBV-HCC patients.

Genotype	No. of Patients	Death (%)	Univariate Analysis		Multivariate Analysis	
			HR (95% CI)	*p*	HR (95% CI)	*p* ^a^
*DCTD* rs17074255 G>A						
GG	300	128 (42.7)	1.00		1.00	
GA	418	210 (50.2)	1.24 (0.99–1.55)	0.056	1.27 (1.02–1.59)	0.033
AA	148	81 (54.7)	1.46 (1.10–1.93)	0.008	1.45 (1.10–1.92)	0.009
Trend				0.003		0.005
GG	300	128 (42.7)	1.00		1.00	
GA + AA	566	291 (51.4)	1.29 (1.05–1.90)	0.016	1.32 (1.07–1.63)	0.010
*ENTPD2* rs3763662 G>A						
GG	315	145 (46.0)	1.00		1.00	
AG	396	196 (49.5)	1.21 (0.97–1.50)	0.086	1.24(1.00–1.54)	0.048
AA	155	78 (50.3)	1.28 (0.97–1.69)	0.076	1.38 (1.05–1.83)	0.023
Trend test				0.036		0.013
GG	315	145 (46.0)	1.00		1.00	
AG + AA	551	274 (50.6)	1.23 (1.00–1.50)	0.046	1.28 (1.05–1.57)	0.017
NUG ^b^						
0	95	39 (41.1)	1.00		1.00	
1	425	195 (45.9)	1.44 (1.01–2.04)	0.042	1.51 (1.06–2.16)	0.022
2	346	185 (53.5)	1.72 (1.21–2.45)	0.002	1.85 (1.30–2.64)	<0.001
Trend test				<0.001		<0.001
0	95	39 (41.1)	1.00		1.00	
1–2	771	380 (49.3)	1.56 (1.12–2.19)	0.009	1.67 (1.19–2.34)	0.003

Abbreviations: SNP: single-nucleotide polymorphism; HBV: hepatitis B virus; HCC: hepatocellular carcinoma; HR: hazard ratio; CI: confidence interval; NUG: number of unfavorable genotypes. ^a^ Multivariate Cox proportional hazards regression analysis was adjusted for age, sex, smoking status, drinking status, AFP level, cirrhosis, embolus, and BCLC stage. ^b^ Unfavorable genotypes were defined der the dominant model as GA + AA for *DCTD* rs17074255 and GA + AA for *ENTPD2* rs3763662 (with GG as the reference), as the A allele was the risk/unfavorable allele for both SNPs. The NUG ranges from 0 to 2 and corresponds to the GRS (genetic risk score) used in the text.

## Data Availability

The data presented in this study are available on request from the corresponding author due to the regulations of the People’s Republic of China on the administration of human genetic resources (State Decree No. 717).

## Supplementary Materials

The following supporting information can be downloaded at https://www.mdpi.com/article/10.3390/cancers18142253/s1, Figure S1: Regional association plots for the two independent SNPs in the nucleotide metabolism pathway genes in the 1000 Genome Project. SNPs in the region of 50 kilobases up or downstream of (A) rs17074255 in *DCTD* with 50 kb up-down-stream of the gene region, (B) rs3763662 in *ENTPD2* with 50 kb up-down-stream of the gene region. Data points are colored according to the level of linkage disequilibrium of each pair of SNPs based on the hg19/1000 Genomes Asian population. The left-hand y-axis shows the association *p*-value of individual SNPs in the HBV-HCC dataset, which is plotted as -log10 (P) against chromosomal base-pair position. The right-hand y-axis shows the recombination rate estimated from HapMap Data Rel 22/phase II JPT and CHB population. **Abbreviations**: SNP, single nucleotide polymorphism; Figure S2: HCC survival prediction of two SNPs by ROC curve. (A) and (B) Three-year and five-year HCC OS prediction by ROC curve. **Abbreviations:** ROC, receiver operating characteristic curve; AUC, area under curve; Figure S3: Differential mRNA expression analysis and overall survival analysis of *DCTD* and *ENTPD2* in 103 HCC cases. Higher expression levels of *DCTD* (A) and *ENTPD2* (B) was correlated with poorer survival. The expression levels were grouped by median value; Figure S4: Differential mRNA expression analysis and overall survival analysis of *DCTD* and *ENTPD2* in HCC from the KM-plot database. Higher expression levels of *ENTPD2* (A) were correlated with poorer survival while the opposite trend was observed in *DCTD* (B). Higher expression levels of *DCTD* were correlated with poorer survival in women’s HCC (C). Figure S5: Mutation frequency of *DCTD* and *ENTPD2* in hepatocellular carcinoma. Mutation frequency of *DCTD* (A) and *ENTPD2* (B) in hepatocellular carcinoma using the online database of the cBioPortal for Cancer Genomics (http://www.cbioportal.org/); Figure S6: Correlation of *ENTPD2* and *DCTD* mRNA expression with regulatory T‑cell (Treg) infiltration in HCC. Scatter plots showing the association between gene expression and Treg infiltration estimated by multiple immune‑deconvolution algorithms (CIBERSORT, CIBERSORT‑ABS, and QUANTISEQ) using publicly available LIHC transcriptomic data. (**A**,**C**,**E**) *ENTPD2* mRNA expression; (**B**,**D**,**F**) *DCTD* mRNA expression. Statistical significance was assessed using Spearman’s rank correlation test. **Abbreviation:** Treg, regulatory T cell; LIHC, liver hepatocellular carcinoma; Table S1: Genes involved in nucleotides metabolism pathway from Gene Ontology Biological Process (MSIGDB). ^a^ Genes were selected based on https://www.gsea-msigdb.org/gsea/msigdb/search.jsp; ^b^ Duplicated genes, pseudogene and genes in X chromosome had been removed; Table S2: The fragments of the *ENTPD2* gene cloned into the luciferase reporter pGL3-promoter vector. ^a^ NCBI, https://www.ncbi.nlm.nih.gov/; Table S3: Associations of demographics and clinical characteristics with overall survival in 866 HBV-HCC patients. **Abbreviations**: No. of patients: number of patients; HBV: hepatitis B virus; HCC: hepatocellular carcinoma. ^a^ Multivariate Cox proportional hazards regression analysis was adjusted for age, sex, smoking status, drinking status, AFP level, cirrhosis, embolus, and BCLC stage; Table S4: Associations of 47 significant SNPs with overall survival of HBV-HCC patients and their eQTL *p*-values in liver and whole blood. **Abbreviations**: SNPs, single nucleotide polymorphisms; HBV, hepatitis B virus; HCC, hepatocellular carcinoma; MAF, Minor Allele Frequency; HR, hazard ratio; 95% CI, 95% confidence interval; BFDP, Bayesian false-discovery probability; eQTL, expression quantitative trait loci; GTEx, Genotype-Tissue Expression. ^a^ Referring allele/effect allele. ^b^ Multivariate Cox proportional hazards regression analysis was adjusted for age, sex, smoking status, drinking status, AFP level, cirrhosis, embolus, and BCLC stage; Table S5: Stratified analysis of combined unfavorable alleles with OS of HBV-related HCC. **Abbreviations**: OS, overall survival; HBV, hepatitis B virus; HCC, hepatocellular carcinoma; NUG, number of unfavorable genotypes; HR, hazard ratio; 95% CI, 95% confidence interval; *p*-inter, *p*-value for interaction. ^a^ Multivariate Cox proportional hazards regression analysis was adjusted for age, sex, smoking status, drinking status, AFP level, cirrhosis, embolus, and BCLC stage. ^b^ *p*-value for multiplicative interaction analysis between variables and NUG.
