# Supplementary material for: Potentially Functional Variants of DCTD and ENTPD2 in the Metabolism of Nucleotide Pathway Genes Predict Survival of HBV-Related Hepatocellular Carcinoma Patients
Source: Cancers (Basel). 2026 Jul 14;18(14):2253. doi: 10.3390/cancers18142253 (PMC13407333; doi:10.3390/cancers18142253)
Supplement: Supplementary file 1 [file cancers-18-02253-s001.zip › cancers-4385358-Supplementary Materials.pdf]

Potentially functional variants of *DCTD* and *ENTPD2* in the metabolism of nucleotides pathway genes predict survival of HBV-related hepatocellular carcinoma patients

List of Supplementary Information:

Figure S1. Regional association plots for the two independent SNPs in the nucleotide metabolism pathway genes in the 1000 Genome Project.

Figure S2. Prediction of survival with *DCTD* rs17074255 and *ENTPD2* rs3763662 and HCC survival prediction of two SNPs by ROC curve.

Figure S3. The association of mRNA expression of *DCTD* and *ENTPD2* with the overall survival of in 103 HCC patients.

Figure S4. The association of mRNA expression of *DCTD* and *ENTPD2* with the overall survival of HCC from KM-plot database

Figure S5. Mutation frequency of *DCTD* and *ENTPD2* in hepatocellular carcinoma

Figure S6. Correlation of *DCTD* and *ENTPD2* mRNA expression with regulatory T-cell (Treg) infiltration in HCC.

Table S1. Genes involved in nucleotides metabolism pathway from Gene Ontology Biological Process (MSIGDB)

Table S2. Two fragments of the *ENTPD2* gene cloned into the luciferase reporter pGL3-promoter vector

Table S3. Associations of demographics and clinical characteristics with overall survival in 866 HBV-HCC patients

Table S4. Associations of 47 significant SNPs with overall survival of HBV-HCC patients and their eQTL *p*-values in liver and whole blood

Table S5. Stratified analysis of combined protective alleles with overall survival of HBV-HCC patients

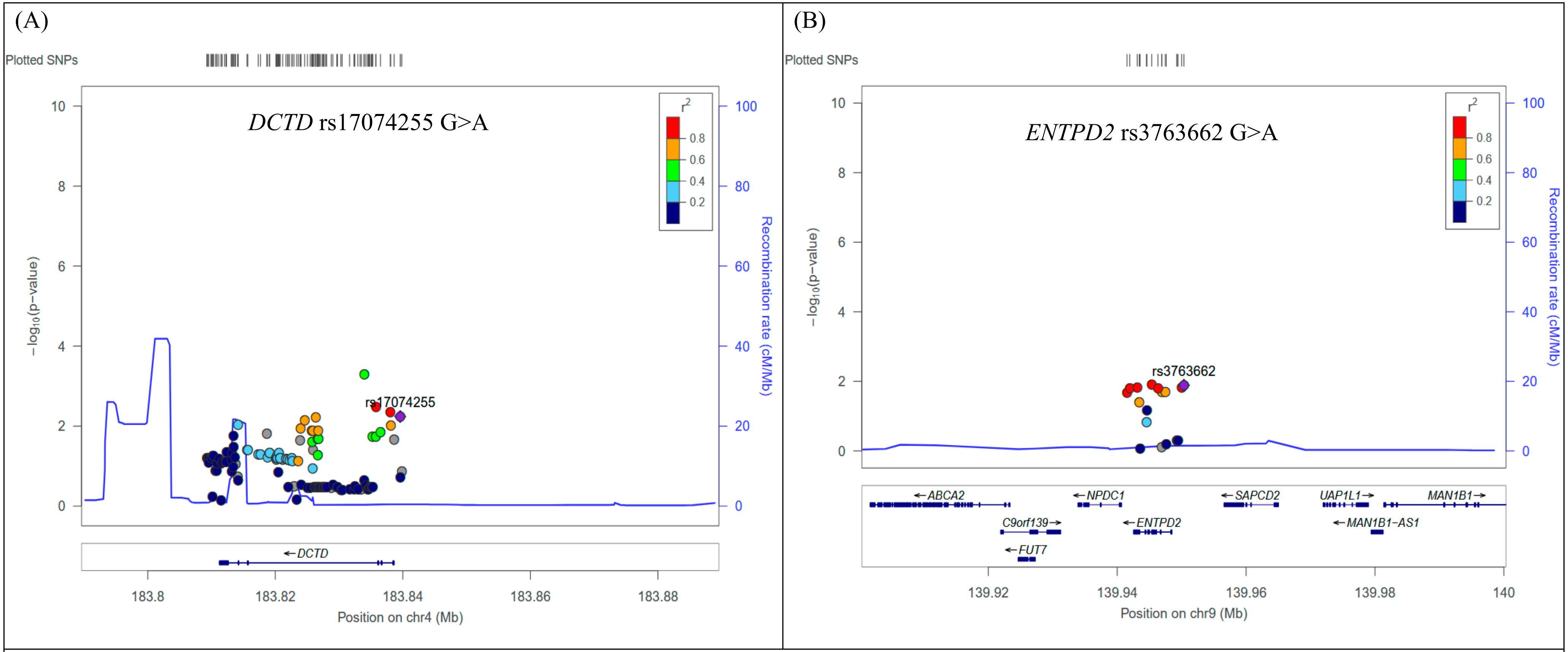

Figure S1. Regional association plots for the two independent SNPs in the nucleotide metabolism pathway genes in the 1000 Genome Project.

SNPs in the region of 50 kilobases up or downstream of (**A**) rs17074255 in *DCTD* with 50 kb up-down-stream of the gene region, (**B**) rs3763662 in *ENTPD2* with 50 kb up-down-stream of the gene region. Data points are colored according to the level of linkage disequilibrium of each pair of SNPs based on the hg19/1000 Genomes Asian population. The left-hand y-axis shows the association P-value of individual SNPs in the HBV-HCC dataset, which is plotted as -log10 (P) against chromosomal base-pair position. The right-hand y-axis shows the recombination rate estimated from HapMap Data Rel 22/phase II JPT and CHB population.

Abbreviations: SNP, single nucleotide polymorphism.

(A)

(B)

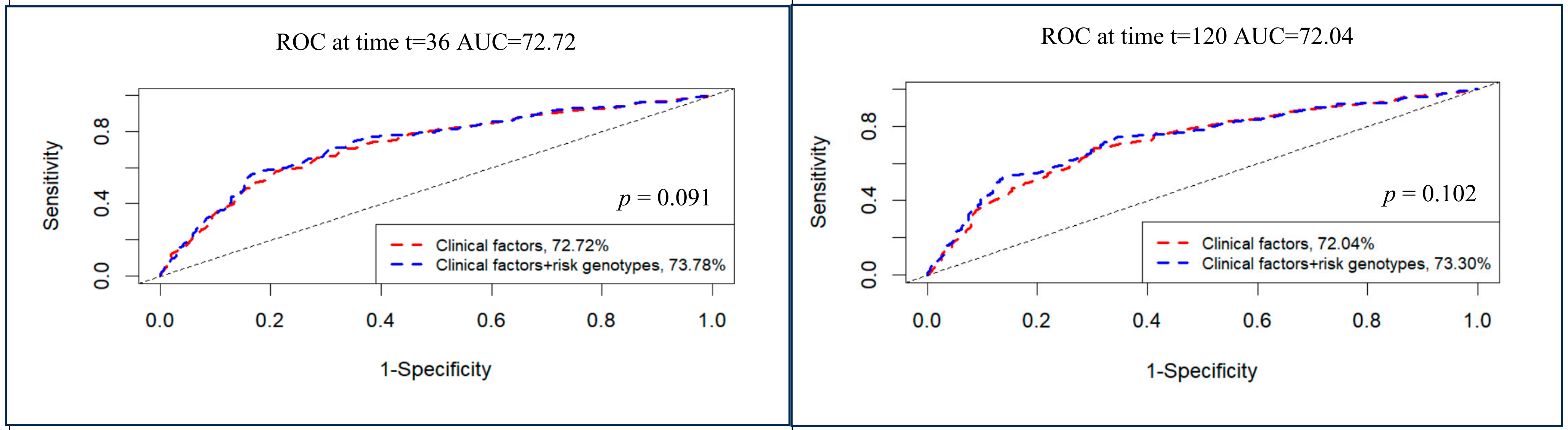

Figure S2. HCC survival prediction of two SNPs by ROC curve. (**A**) and (**B**) Three-year and five-year HCC OS prediction by ROC curve.
Abbreviations: ROC, receiver operating characteristic curve; AUC, area under curve.

(A)

(B)

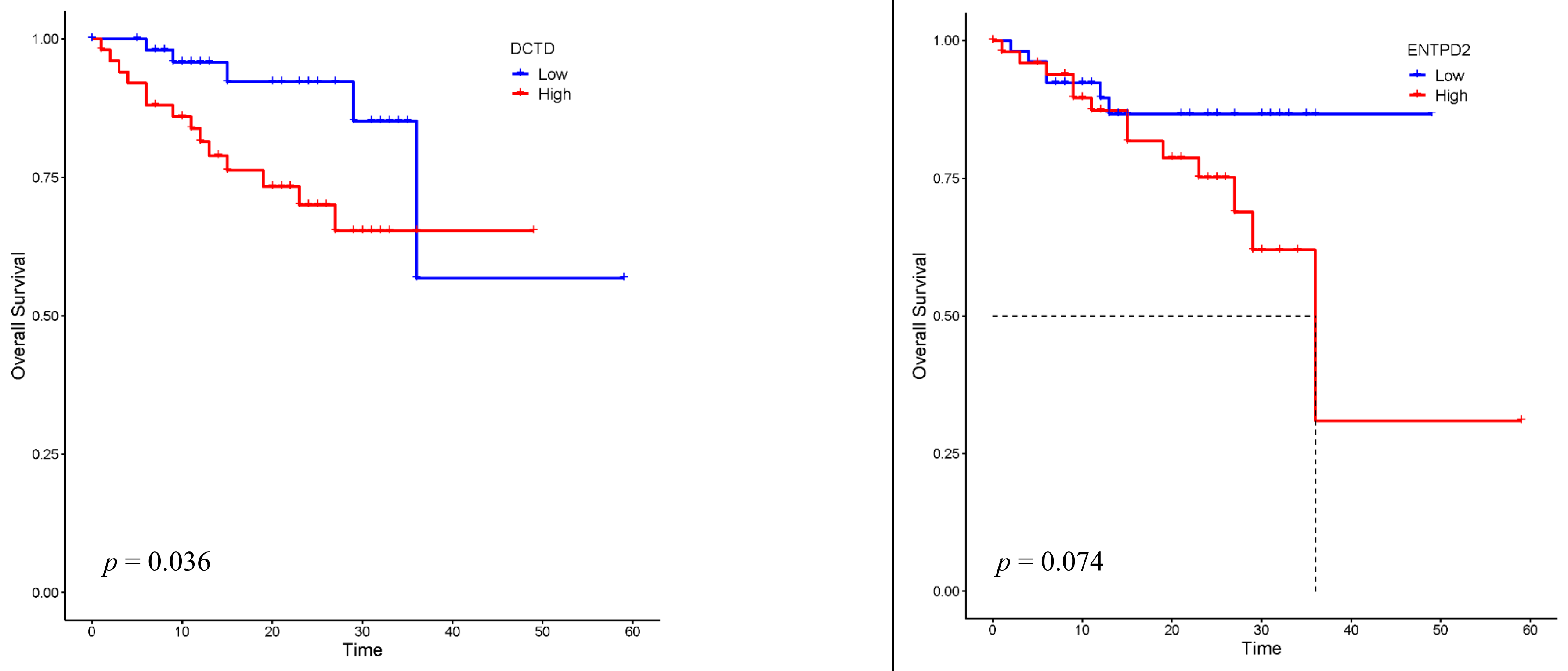

Figure S3. Differential mRNA expression analysis and overall survival analysis of *DCTD* and *ENTPD2* in 103 HCC cases.

Higher expression levels of *DCTD* (**A**) and *ENTPD2* (**B**) was correlated with poorer survival. The expression levels were grouped by median value.

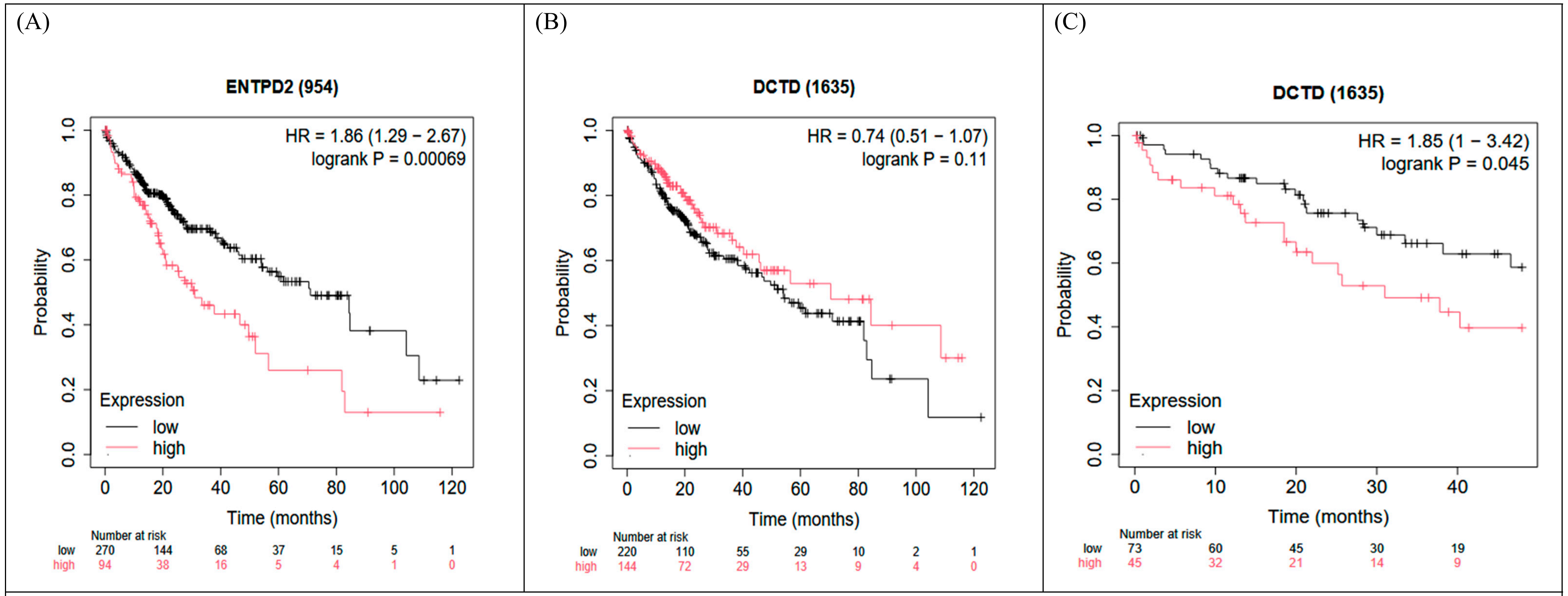

Figure S4. Differential mRNA expression analysis and overall survival analysis of *DCTD* and *ENTPD2* in HCC from the KM-plot database. Higher expression levels of *ENTPD2* (**A)** were correlated with poorer survival while the opposite trend was observed in *DCTD* (**B**). Higher expression levels of *DCTD* were correlated with poorer survival in women's HCC (**C**).

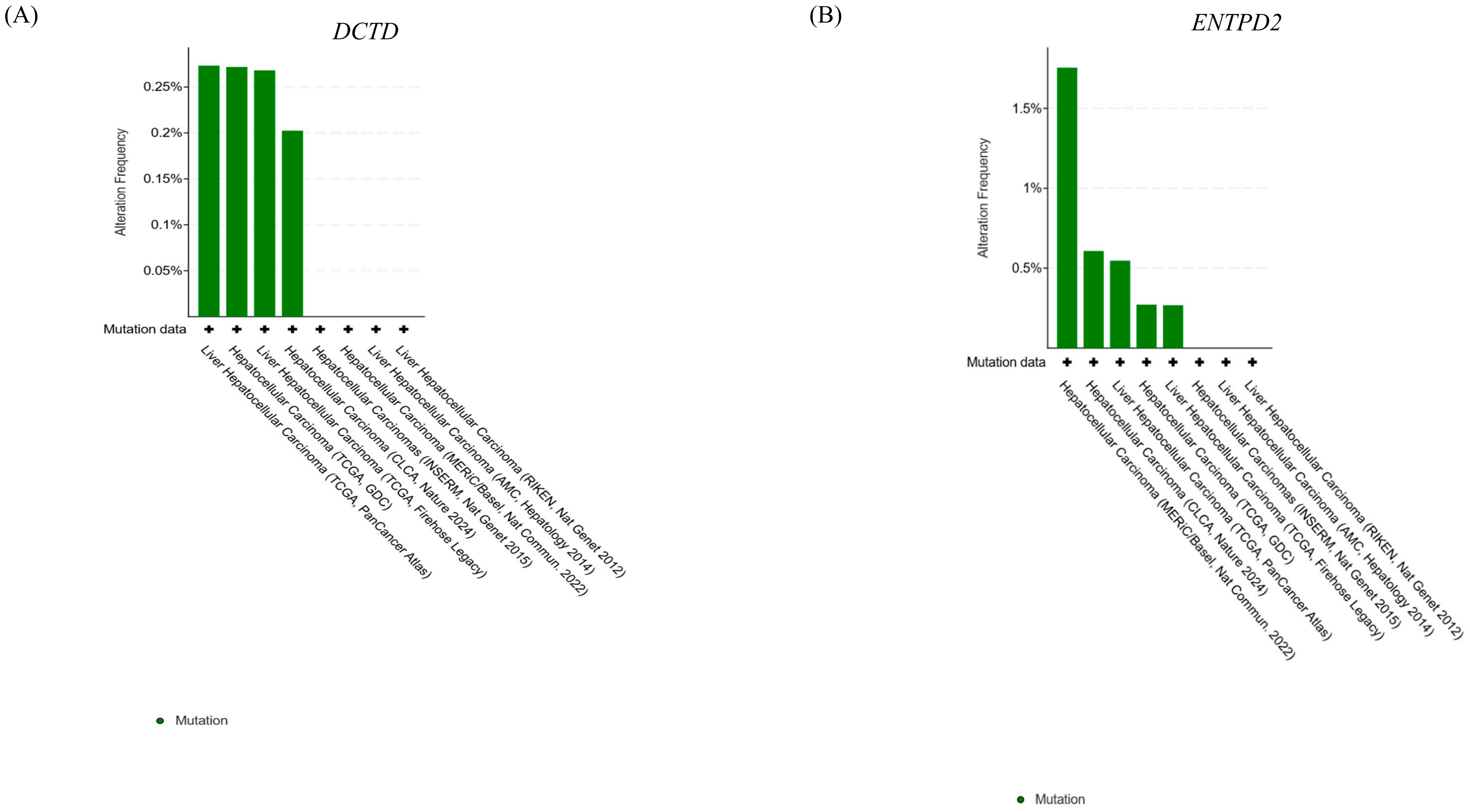

Figure S5. Mutation frequency of *DCTD* and *ENTPD2* in hepatocellular carcinoma.

Mutation frequency of *DCTD* (**A**) and *ENTPD2* (**B**) in hepatocellular carcinoma using the online database of the cBioPortal for Cancer Genomics (http://www.cbioportal.org/).

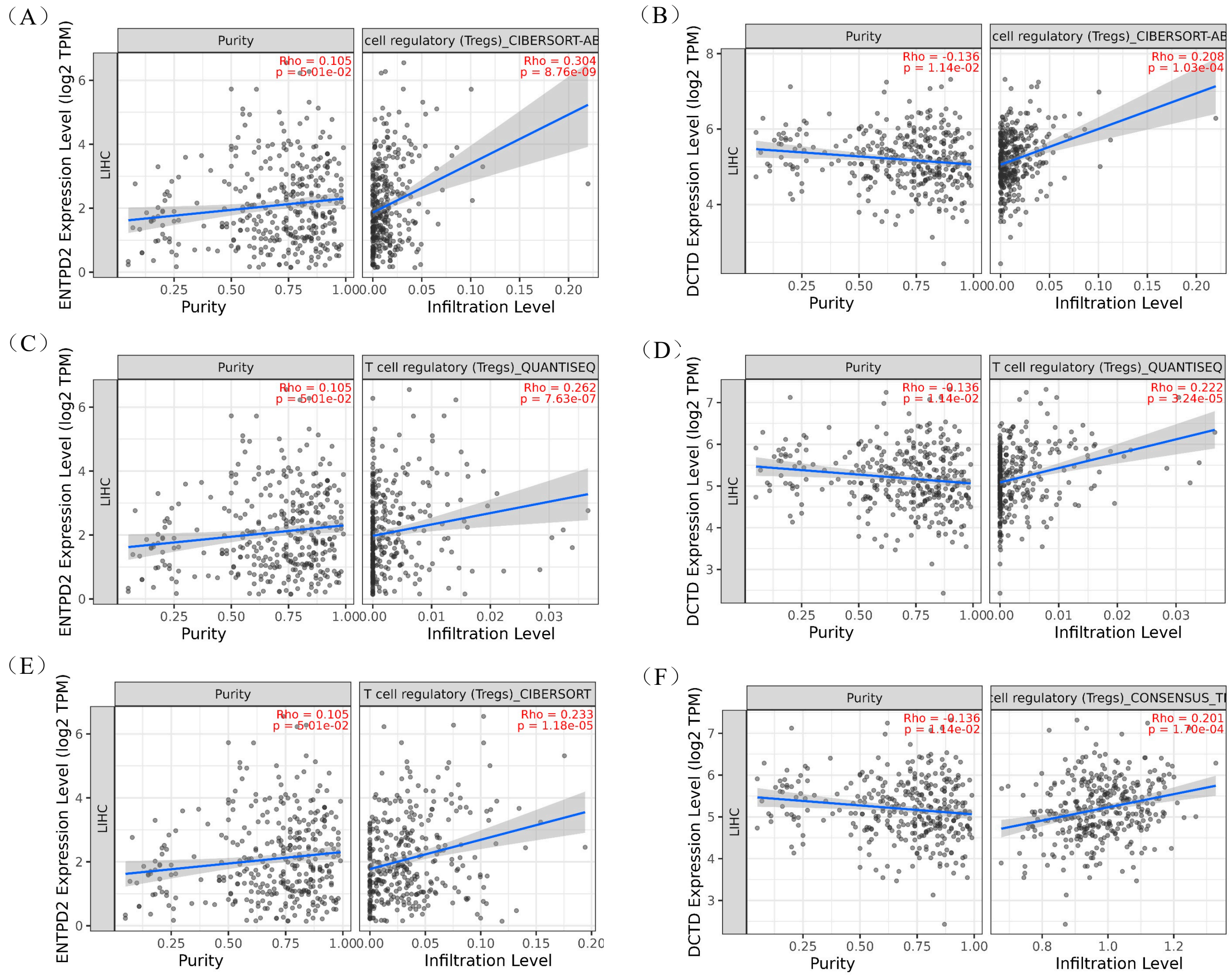

Figure S6. Correlation of *ENTPD2* and *DCTD* mRNA expression with Treg infiltration in HCC.

Scatter plots showing the association between gene expression and Treg infiltration estimated by multiple immune-deconvolution algorithms (CIBERSORT, CIBERSORT-ABS, and QUANTISEQ) using publicly available LIHC transcriptomic data. **(A, C, E)** *ENTPD2* mRNA expression; **(B, D, F)** *DCTD* mRNA expression. Statistical significance was assessed using Spearman's rank correlation test.

Abbreviation: Treg, regulatory T cell; LIHC, liver hepatocellular carcinoma.

Table S1. Genes involved in nucleotides metabolism pathway from Gene Ontology Biological Process (MSIGDB)

Geneset	Selected genes [a]	Number of genes
STANDARD_NAME REACTOME_METABOLISM_OF_NUCLEOTIDES	*AK2, UPP2, TYMP, ADSS2, RRM2B, ENTPD2, NT5C2, CAD, AK6, UPB1, GMPR2, SAMHD1, DHODH, NME3, NME4, GSR, NUDT1, IMPDH1, AK1, DNPH1, AGXT2, UMPS, DGUOK, AMPD2 AMPD1, NT5C1A, GDA, NT5C3A, NT5C, ITPA, PAICS, PPAT, DUT, DCTD, UCK1, AMPD3, NT5E, NUDT15, TXN, GMPR, ENTPD1, ATIC, AK7, UCK2, GUK1, DPYS, AK5, AK9, ADK, DCK, XDH, CDA, GART, CMPK1, AK4, GMPS, NUDT5, AK8, NUDT13, TK2, RRM1, TK1, ENTPD3, DTYMK, ADAL, ADPRM, NUDT9, CTPS1, RRM2, GLRX, TYMS, IMPDH2, PFAS, DCTPP1, UPP1, NT5C1B, ADSS1, ENTPD5, DPYD, ENTPD8, ADA, ENTPD4 ENTPD6, ENTPD7, UCKL1, TXNRD1, NUDT16, PNP, APRT, NT5M, NME1, ADSL, NME2, NUDT*18	58
Gene knockout	*CTPS2, PUDP* and *HPRT1* are located on the X chromosome	3
Total genes		94 [b]

[a] Genes were selected based on https://www.gsea-msigdb.org/gsea/msigdb/search.jsp;

[b] Duplicated genes, pseudogene and genes in X chromosome had been removed.

Table S2. The fragments of the *ENTPD2* gene cloned into the luciferase reporter pGL3-promoter vector

Haplotypes	Base sequence [a]
rs3763662 G (reference)	GGTACCcgcccggcccacaaccggctaatttttgtatttctattagagacggcgtttcaccatgttggccaggctggtctt<br>gaactcctgaccttaagtgatccacctgcctcagcttcccaaagtgctggaattacagacgtgagccacacagcacaagctt<br>ctgatgtggagactgaggcagggcagccaagatgtcagcaggcctcaggaaagccccccctccctgggaggtctgagccc<br>tgcgtgggcgggaggctccgagttccctcccccaggggttctcccctaacctctctgcatccgcatgttcccagctcagccg<br>gggtcatgtgcagcacatctggaggccccatccccgagagggtcctggaagccgagagctgccccacccagaggcccct<br>ccaccccttcagctcctgccagcttccctctgacgctctcccaagtctgccttccagggtccctgagtccatgccaggaagg<br>ggcgttcccccgccctcactccgtctcaaagagagctcagcctcagcctttgtcctggccgctctgggctccctgcctctccc<br>tccgcaggcgaagctgggcactcggccatgagcctgtctccccggggcagagtccagctcccctctccctccccatcctct<br>caggcctggcagacctcccactccctggcttgagctttttgtccatgaaaagagtccagaacaccaggaagccccgctggg<br>cctggagggaagcggggtaccgcaggccagtgggggtcgcagcaccctgcctctgggcggcctttgttccttccttttcag<br>gcatgatgtgcactgtgcgtgcctctccctcctctgggcggtcaggggtggaagaaaggcctggggtgtgtgggggggaaa<br>ctggggcctggggcctggagcttagcaccggcaacacccagagattgcctgctggagccccgccaggctgggctgtgag<br>gctgaggcagcagagaggcttcccagggttcccaccAAGCTT
rs3763662 A (alter)	GGTACCcgcccggcccacaaccggctaatttttgtatttctattagagacggcgtttcaccatgttggccaggctggtctt<br>gaactcctgaccttaagtgatccacctgcctcagcttcccaaagtgctggaattacagacgtgagccacacagcacaagctt<br>ctgatgtggagactgaggcagggcagccaagatgtcagcaggcctcaggaaagccccccctccctgggaggtctgagccc<br>tgcgtgggcgggaggctccgagttccctcccccaggggttctcccctaacctctctgcatccgcatgttcccagctcagccg<br>gggtcatgtgcagcacatctggaggccccatccccgagagggtcctggaagccgagagctgccccacccagaggcccct<br>ccaccccttcagctcctgccagcttccctctgacgctctcccaagtctgccttccagggtccctgagtccatgccaggaagg<br>ggcgttcccccgccctcactcAgtctcaaagagagctcagcctcagcctttgtcctggccgctctgggctccctgcctctcc<br>ctccgcaggcgaagctgggcactcggccatgagcctgtctccccggggcagagtccagctcccctctccctccccatcctc<br>tcaggcctggcagacctcccactccctggcttgagctttttgtccatgaaaagagtccagaacaccaggaagccccgctgg<br>gcctggagggaagcggggtaccgcaggccagtgggggtcgcagcaccctgcctctgggcggcctttgttccttccttttca<br>ggcatgatgtgcactgtgcgtgcctctccctcctctgggcggtcaggggtggaagaaaggcctggggtgtgtgggggggaa<br>actggggcctggggcctggagcttagcaccggcaacacccagagattgcctgctggagccccgccaggctgggctgtga<br>ggctgaggcagcagagaggcttcccagggttcccaccAAGCTT

[a] NCBI, https://www.ncbi.nlm.nih.gov/

Table S3. Associations of demographics and clinical characteristics with overall survival in 866 HBV-HCC patients

Characteristics	No. of patients (%)	Death (%)	Univariable analysis		Multivariable analysis	
			HR (95% CI)	*p*	HR (95% CI)	*p* [a]
Age (year)						
≤47	434	233 (53.7)	1.00		1.00	
>47	432	186 (43.1)	0.72 (0.59-0.87)	<0.001	0.81 (0.66-0.99)	0.036
Sex						
Female	106	42 (39.6)	1.00		1.00	
Male	760	377 (49.6)	1.25 (0.90-1.71)	0.179	1.26 (0.90-1.76)	0.176
Smoking status						
No	545	268 (49.2)	1.00		1.00	
Yes	321	151 (47.0)	0.96 (0.79-1.18)	0.724	0.91 (0.71-1.17)	0.475
Drinking status						
No	614	292 (47.6)	1.00		1.00	
Yes	252	127 (50.4)	1.07 (0.87-1.32)	0.500	1.08 (0.84-1.41)	0.541
AFP (ng/mL)						
≤400	522	232 (44.4)	1.00		1.00	
>400	344	187 (54.4)	1.59 (1.31-1.93)	<0.001	1.29 (1.05-1.57)	0.015
Cirrhosis						
No	390	184 (47.2)	1.00		1.00	
Yes	476	235 (49.4)	1.00 (0.82-1.22)	0.996	1.04 (0.85-1.26)	0.702
Cancer embolus						
No	636	260 (40.1)	1.00		1.00	
Yes	230	159 (69.1)	2.73 (2.32-3.34)	<0.001	1.74 (1.38-2.21)	<0.001
BCLC stage						
0/A	427	146 (34.2)	1.00		1.00	
B/C	439	273 (62.2)	2.73 (2.23-3.35)	<0.001	1.98 (1.56-2.52)	<0.001

Abbreviations: No. of patients: number of patients; HBV: hepatitis B virus; HCC: hepatocellular carcinoma.

[a] Multivariate Cox proportional hazards regression analysis was adjusted for age, sex, smoking status, drinking status, AFP level, cirrhosis, embolus, and BCLC stage

Table S4. Associations of 47 significant SNPs with overall survival of HBV-HCC patients and their eQTL *p*-values in liver and whole blood

SNPs	Chr	Gene	Allele [a]	MAF	HR (95% CI)	*p* [b]	BFDP	FPRP	eQTL-GTEx	
									p-liver	*p*-whole_blood
rs532545	1	*CDA*	C>T	0.09	1.33(1.04-1.7)	0.023	0.729	0.198	6.10×10^{-2}	2.00×10^{-32}
rs602950	1	*CDA*	A>G	0.09	1.35(1.06-1.71)	0.013	0.635	0.125	6.30×10^{-2}	1.40×10^{-32}
rs2072671	1	*CDA*	A>C	0.09	1.35(1.06-1.71)	0.013	0.635	0.125	4.60×10^{-1}	3.80×10^{-34}
rs7555294	1	*DPYD*	C>T	0.29	1.21(1.04-1.42)	0.014	0.751	0.15	3.10×10^{-2}	2.40×10^{-4}
rs1879375	1	*DPYD*	A>G	0.30	1.25(1.08-1.46)	0.003	0.485	0.042	1.50×10^{-1}	1.70×10^{-4}
rs12046771	1	*DPYD*	T>C	0.31	1.25(1.07-1.45)	0.004	0.404	0.028	1.10×10^{-1}	2.10×10^{-5}
rs10875071	1	*DPYD*	T>C	0.31	1.25(1.07-1.45)	0.004	0.404	0.028	1.10×10^{-1}	2.60×10^{-5}
rs12487941	3	*UMPS*	C>T	0.10	1.28(1.04-1.58)	0.020	0.734	0.173	3.10×10^{-1}	1.10×10^{-4}
rs113871220	3	*UMPS*	G>A	0.10	1.28(1.04-1.58)	0.020	0.734	0.173	3.10×10^{-1}	1.10×10^{-4}
rs12492095	3	*UMPS*	T>A	0.10	1.28(1.04-1.58)	0.020	0.734	0.173	3.10×10^{-1}	1.30×10^{-4}
rs1440148	3	*UMPS*	G>A	0.10	1.28(1.04-1.58)	0.020	0.734	0.173	3.10×10^{-1}	1.30×10^{-4}
rs2279197	3	*UMPS*	G>A	0.10	1.28(1.04-1.58)	0.020	0.734	0.173	3.10×10^{-1}	9.90×10^{-5}
rs9840078	3	*UMPS*	G>T	0.10	1.28(1.04-1.58)	0.020	0.734	0.173	3.10×10^{-1}	1.30×10^{-4}
rs9844948	3	*UMPS*	C>A	0.10	1.28(1.04-1.58)	0.020	0.734	0.173	3.10×10^{-1}	1.30×10^{-4}
rs10934682	3	*UMPS*	T>G	0.10	1.28(1.04-1.57)	0.022	0.706	0.146	3.10×10^{-1}	1.30×10^{-4}
rs9870260	3	*UMPS*	T>A	0.10	1.28(1.04-1.57)	0.022	0.706	0.146	3.10×10^{-1}	1.30×10^{-4}
rs13092695	3	*UMPS*	T>C	0.10	1.28(1.04-1.57)	0.022	0.706	0.146	3.10×10^{-1}	1.30×10^{-4}
rs1966981	3	*UMPS*	C>T	0.10	1.28(1.04-1.57)	0.022	0.706	0.146	3.10×10^{-1}	1.30×10^{-4}
rs16835929	3	*UMPS*	A>G	0.10	1.28(1.04-1.57)	0.022	0.706	0.146	3.00×10^{-1}	$6.00E \times 10^{-5}$
rs1801019	3	*UMPS*	G>C	0.10	1.28(1.04-1.57)	0.022	0.706	0.146	3.10×10^{-1}	9.00×10^{-5}
rs13061561	3	*UMPS*	C>T	0.10	1.28(1.04-1.57)	0.022	0.706	0.146	3.10×10^{-1}	1.30×10^{-4}
rs17843826	3	*UMPS*	T>G	0.10	1.28(1.04-1.57)	0.022	0.706	0.146	3.10×10^{-1}	1.30×10^{-4}
rs3772806	3	*UMPS*	C>T	0.10	1.28(1.04-1.57)	0.022	0.706	0.146	2.00×10^{-1}	2.20×10^{-4}
rs4678148	3	*UMPS*	C>T	0.10	1.28(1.04-1.57)	0.022	0.706	0.146	3.10×10^{-1}	9.00×10^{-5}
rs4678149	3	*UMPS*	T>G	0.10	1.28(1.04-1.57)	0.022	0.706	0.146	3.10×10^{-1}	9.00×10^{-5}
rs9875527	3	*UMPS*	C>T	0.10	1.28(1.04-1.57)	0.022	0.706	0.146	3.10×10^{-1}	9.00×10^{-5}
rs13146	3	*UMPS*	C>T	0.10	1.28(1.04-1.57)	0.022	0.706	0.146	3.10×10^{-1}	9.00×10^{-5}
rs2242247	3	*UMPS*	A>T	0.10	1.28(1.04-1.57)	0.022	0.706	0.146	3.10×10^{-1}	9.00×10^{-5}
rs2242248	3	*UMPS*	G>T	0.10	1.28(1.04-1.57)	0.022	0.706	0.146	3.10×10^{-1}	9.00×10^{-5}
rs4677940	3	*UMPS*	G>A	0.10	1.28(1.04-1.57)	0.022	0.706	0.146	3.10×10^{-1}	1.70×10^{-4}
rs1979411	3	*UMPS*	A>G	0.10	1.28(1.04-1.57)	0.022	0.706	0.146	3.10×10^{-1}	2.10×10^{-4}
rs1979412	3	*UMPS*	G>A	0.10	1.28(1.04-1.57)	0.022	0.706	0.146	3.10×10^{-1}	2.40×10^{-4}
rs1979413	3	*UMPS*	G>A	0.10	1.28(1.04-1.57)	0.022	0.706	0.146	3.10×10^{-1}	2.10×10^{-4}
rs12497347	3	*UMPS*	C>T	0.10	1.28(1.04-1.57)	0.022	0.706	0.146	3.10×10^{-1}	2.10×10^{-4}
rs12487906	3	*UMPS*	T>C	0.10	1.28(1.04-1.58)	0.021	0.734	0.173	3.10×10^{-1}	2.10×10^{-4}
**rs17074255**	**4**	***DCTD***	**G>A**	**0.41**	**1.21(1.06-1.39)**	**0.006**	**0.583**	**0.06**	$\mathbf{5.10 \times 10^{-4}}$	$\mathbf{8.60\times 10^{-2}}$
rs6890048	5	*GLRX*	G>C	0.09	0.72(0.55-0.94)	0.015	0.664	0.166	6.50×10^{-1}	7.90×10^{-8}
rs9411300	9	*ENTPD2*	T>A	0.41	1.17(1.02-1.34)	0.021	0.797	0.173	8.50×10^{-4}	1.10×10^{-14}
rs3814504	9	*ENTPD2*	G>C	0.41	1.18(1.03-1.35)	0.016	0.742	0.125	6.50×10^{-4}	1.80×10^{-4}
rs17853460	9	*ENTPD2*	A>G	0.41	1.18(1.03-1.35)	0.015	0.742	0.125	3.10×10^{-4}	9.00×10^{-7}
rs2292925	9	*ENTPD2*	G>A	0.41	1.19(1.04-1.36)	0.012	0.672	0.088	1.10×10^{-4}	1.40×10^{-7}
rs4880084	9	*ENTPD2*	C>T	0.41	1.18(1.03-1.35)	0.016	0.742	0.125	6.50×10^{-5}	2.90×10^{-8}
rs9411242	9	*ENTPD2*	G>A	0.41	1.18(1.03-1.35)	0.015	0.742	0.125	6.50×10^{-5}	2.40×10^{-8}

**rs3763662**	**9**	***ENTPD2***	**G>A**	**0.41**	**1.19(1.04-1.36)**	**0.013**	**0.672**	**0.088**	**9.20×10^{-5}**	**4.80×10^{-8}**
rs535070	16	*APRT*	G>A	0.36	1.17(1.01-1.34)	0.033	0.797	0.173	2.60×10^{-1}	9.10×10^{-4}
rs9953424	18	*ENOSF1*	C>T	0.28	0.8(0.68-0.94)	0.007	0.548	0.057	2.00×10^{-8}	4.70×10^{-4}
rs9948583	18	*ENOSF1*	T>C	0.29	0.83(0.71-0.97)	0.022	0.751	0.147	2.20×10^{-8}	5.50×10^{-4}

Abbreviations: SNPs, single nucleotide polymorphisms; HBV, hepatitis B virus; HCC, hepatocellular carcinoma; MAF, Minor Allele Frequency; HR, hazard ratio; 95% CI, 95% confidence interval; BFDP, Bayesian false-discovery probability; eQTL, expression quantitative trait loci; GTEx, Genotype-Tissue Expression.

[a] Referring allele/effect allele.

[b] Multivariate Cox proportional hazards regression analysis was adjusted for age, sex, smoking status, drinking status, AFP level, cirrhosis, embolus, and BCLC stage.

Table S5. Stratified analysis of combined unfavorable alleles with OS of HBV-related HCC

Characteristics	NUG 0		NUGs 1-2		Multivariate analysis		
	All	Death (%)	All	Death (%)	HR (95% CI)	*p* [a]	*p*-inter [b]
Age (year)							0.312
≤47	47	26 (55.3)	387	207 (53.5)	1.47 (0.95-2.26)	0.08	
>47	48	13 (27.0)	384	173 (45.1)	2.04 (1.15-3.61)	0.014	
Sex							0.188
Female	22	9 (40.9)	84	33 (39.3)	0.73 (0.33-1.64)	0.446	
Male	73	30 (41.1)	687	347 (50.5)	1.98 (1.34-2.92)	<0.001	
Smoking status							0.722
No	61	24 (39.3)	484	244 (50.4)	1.72 (1.11-2.66)	0.015	
Yes	34	15 (44.1)	287	136 (47.4)	1.50 (0.87-2.63)	0.147	
Drinking status							0.873
No	70	28 (40.0)	544	264 (48.5)	1.44 (0.97-2.15)	0.074	
Yes	25	11 (44.0)	227	116 (51.1)	2.66 (1.38-5.15)	0.004	
AFP level (ng/mL)							0.334
≤400	58	24 (41.4)	464	208 (44.8)	1.60 (1.03-2.47)	0.035	
>400	37	15 (40.5)	307	172 (56.0)	1.70 (0.98-2.73)	0.058	
Cirrhosis							0.52
No	43	17 (39.5)	347	167 (48.1)	2.16 (1.29-3.63)	0.005	
Yes	52	22 (42.3)	424	213 (50.2)	1.36 (0.87-2.15)	0.181	
Cancer embolus							0.268
No	69	26 (37.7)	567	234 (41.3)	1.44 (0.94-2.20)	0.091	
Yes	26	13 (50.0)	204	146 (71.6)	2.04 (1.12-3.73)	0.021	
BCLC stage							0.407
0/A	50	15 (30.0)	377	131 (34.7)	1.43 (0.83-1.47)	0.196	
B/C	45	24 (53.3)	394	249 (63.2)	1.73 (1.11-2.68)	0.015	

Abbreviations: OS, overall survival; HBV, hepatitis B virus; HCC, hepatocellular carcinoma; NUG, number of unfavorable genotypes; HR, hazard ratio; 95% CI, 95% confidence interval; *p*-inter, *p*-value for interaction.

[a] Multivariate Cox proportional hazards regression analysis was adjusted for age, sex, smoking status, drinking status, AFP level, cirrhosis, embolus, and BCLC stage.

[b] *p*-value for multiplicative interaction analysis between variables and NUG.
